# Racial, Ethnic, and Color-Based Discrimination and Pre-Pregnancy Risk Factors for Preeclampsia Among Nulliparous Patients

**DOI:** 10.1089/heq.2024.0173

**Published:** 2025-05-23

**Authors:** Alexa I.K. Campbell, Maria J. Small, Sarahn M. Wheeler, Jerome J. Federspiel

**Affiliations:** ^1^ Duke University School of Medicine, Durham, North Carolina, USA.; ^2^ Division of Maternal Fetal Medicine, Department of Obstetrics and Gynecology, Duke University School of Medicine, Durham, North Carolina, USA.; ^3^ Department of Population Health Sciences, Duke University School of Medicine, Durham, North Carolina, USA.; ^4^ Division of Hematology, Department of Medicine, Duke University School of Medicine, Durham, North Carolina, USA.

**Keywords:** racism, obesity, hypertension, preeclampsia, discrimination

## Abstract

**Introduction:**

Obesity and chronic hypertension are well-known risk factors for maternal morbidity and mortality. Evidence suggests racism contributes to the development of these chronic conditions.

**Methods:**

We conducted a secondary analysis of the Nulliparous Pregnancy Outcomes: monitoring mothers-to-be (nuMoM2b) cohort, which recruited nulliparous pregnant participants in the United States in 2010–2013. Using logistic regression, we assessed the relationship between experiences of racial, ethnic, and color-based (REC) discrimination (categorized as high, low, or no REC discrimination) and prevalence of a composite outcome of obesity and/or chronic hypertension.

**Results:**

Among 8,554 participants, the composite outcome was unequally distributed by race and ethnicity (*p* < 0.001), present in 19.9% of non-Hispanic White, 23.1% of Hispanic, and 39.0% of non-Hispanic Black participants. Self-reported REC discrimination was similarly unequally distributed (*p* < 0.001), with high REC discrimination reported by 17.5% of non-Hispanic Black, 10.6% of Hispanic, and 2.1% of and non-Hispanic White participants. In multivariable analyses, high self-reported REC discrimination was associated with a 1.75 adjusted odds ratio (95% confidence interval: 1.43–2.14) of the composite outcome compared with those reporting no REC discrimination. When stratified by race and ethnicity, the odds ratios for the composite outcome among those reporting high REC discrimination were only statistically significant among the Hispanic subgroup.

**Conclusion:**

We observed a positive, dose-dependent association between self-reported REC discrimination and our outcome of obesity and/or chronic hypertension. By demonstrating this relationship in an obstetric cohort, we aim to highlight the role of racism over the life course in contributing to chronic health conditions and associated maternal outcomes.

## Introduction

Chronic hypertension and obesity are two of the most well-documented and prevalent chronic conditions that predispose pregnant people to preeclampsia,^1,2^ an important contributor to maternal morbidity and mortality in the United States.^3^ Notably, chronic hypertension, obesity, and preeclampsia disproportionately impact Black birthing people.^3,4^ Racial and ethnic disparities in preeclampsia extend beyond the non-Hispanic Black population, although less definitively. For birthing Hispanic people, research demonstrates mixed findings of elevated or similar risk for preeclampsia when compared with their non-Hispanic White counterparts.^5^

Race is a social construct, not a biological characteristic, and racism is increasingly recognized as driving racial health disparities.^6^ One mechanism through which racism may act is increased allostatic load, or the physiological effect of racism due to compounded stress responses over a lifetime.^7,8^ Experiences of racial discrimination are a measurable contributor to allostatic load. Both high allostatic load and experiences of discrimination are associated with poor health outcomes, including obesity and chronic hypertension.^7,9,10^ While this understanding of causal pathways is well understood by disparities research, in applied research work, these chronic conditions are often treated as confounding variables in research of associations between race or racism and pregnancy outcomes such as preeclampsia.^11^ Doing so likely falsely attenuates the relationship between racism and pregnancy outcomes by blocking one for the pathways through which racism impacts health.^12^ We aimed to evaluate the association between self-reported experiences of racial, ethnic, and color-based (REC) discrimination and chronic hypertension and obesity prevalence in an obstetric cohort, hypothesizing a positive correlation between these variables.

## Methods

### Study design

We conducted a secondary analysis of the Nulliparous Pregnancy Outcomes Study: Monitoring Mothers-to-be (nuMoM2b) Cohort, a multicenter, prospective study conducted between 2010 and 2015, with previously described methods.^13^ The cohort included approximately 10,000 nulliparous pregnant people recruited and followed throughout pregnancy from eight academic centers across the United States (California, Indiana, Illinois, New York, Ohio, Pennsylvania, and Utah). Comprehensive data were collected via three study visits and chart review, including demographic characteristics, clinical outcomes, psychosocial measures, and social determinants of health (SDOH or non-medical conditions that affect a person’s health, well-being, and quality of life).

**FIG. 1.**
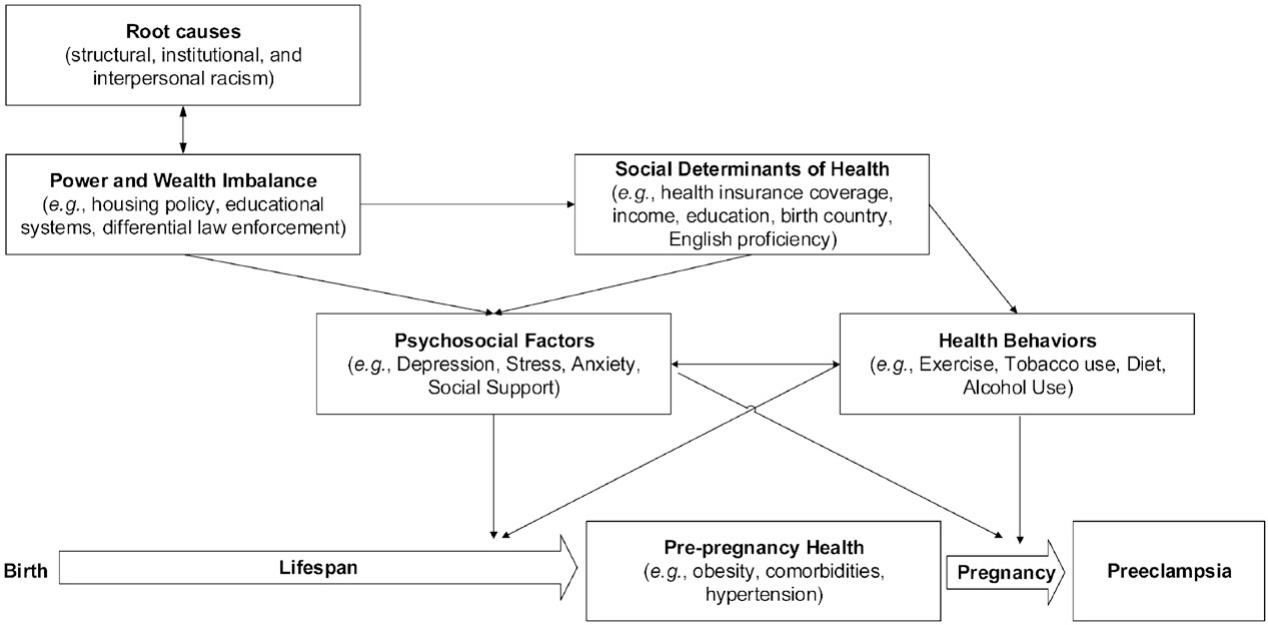
Conceptual model of preeclampsia. Developed based on a theoretical model published by the Society for Maternal Fetal Medicine,^14^ this model emphasizes the downstream consequences of racism and subsequent power and wealth imbalances on individual health over the life course and in pregnancy, specifically in preeclampsia development. This figure is not meant to be comprehensive—it is limited to patient-level characteristics and those variables as measured within nuMoM2b and excludes most structural and environmental variables that may influence preeclampsia.

To guide our analysis, we developed a theoretical model of how chronic conditions contribute to preeclampsia development (Fig. 1), adapted from a model published by the Society for Maternal Fetal Medicine,^14^ itself adapted from Hofrichter and Bhatia^15^ and Crear-Perry.^16^ We maintain the model’s basic structure and add a lifespan perspective, demonstrating how root causes and their downstream consequences influence pregnancy outcomes in the pre-pregnancy and pregnancy periods.^17^ The theoretical model includes variables in the nuMoM2b dataset, yielding a study-specific conceptual model of patient-level variables that contribute to chronic health conditions and preeclampsia. Given that the dataset lacks health systems or other environmental variables, the theoretical model is limited to patient-level characteristics.

We engaged two community groups, Born in Durham, Healthy for Life (BIDHFL) and Latinx Advocacy Team & Interdisciplinary Network for COVID-19 (LATIN-19), both composed of local stakeholders related to each group’s mission (see Supplementary Table S2 for represented stakeholders). Before data analysis, we presented our theoretical model and analytic plan to BIDHFL, a group focused on perinatal equity that is made up of community-based organizations, advocacy groups, clinical leaders, and other stakeholders. Based on this consultation, we chose self-reported experiences of REC discrimination as the explanatory variable. BIDHFL also helped select chronic hypertension and obesity as our composite outcome variable. After the initial analysis, we presented results to BIDHFL, who assisted with their interpretation. Based on our preliminary findings, we also consulted LATIN-19, a multidisciplinary group of stakeholders in the Latinx community, whose initial work on COVID-19 health disparities has expanded into improving health in the Latinx community more broadly.^18^ Their mission includes collaboration to address health disparities, engaging marginalized communities, and promoting research. We presented our preliminary findings to LATIN-19 members, and they assisted with their interpretation through open discussion, specifically providing insight regarding the Latinx population and related limitations of categorization in the data.

### Variables

The explanatory variable, REC discrimination, was measured by an adapted version of the Experiences of Discrimination Scale, a validated measure of self-reported discrimination.^19^ The adaptation used for the nuMoM2b study asks participants whether they have experienced discrimination because of race, ethnicity, or color in nine different settings, such as at school, at work, or receiving medical care. Scores can range from 0 to 9, with scores of 3+ generally categorized as high discrimination.^19^

Participants self-reported race and ethnicity. For the analysis, participants were categorized by race and ethnicity groups as per the Office of Management and Budget standards (e.g., non-Hispanic Black, non-Hispanic White, Hispanic, and Asian) according to their self-reported information. This categorization uses the term “Hispanic,” which we correspondingly use; however, we acknowledge that individuals included in this category may self-identify with other terms (e.g., Latinx). Regarding insurance coverage, participants selected all applicable methods for paying health care expenses, including governmental, commercial, military, self-pay, or other coverage. This variable was recoded into mutually exclusive categories, which defined participants by whichever payment method they specified that typically provides the most expansive coverage. Participants with a history of living outside the United States were defined as those reporting having lived outside of the United States for one year or more.

Obesity was measured at the first trimester study visit and defined as a body mass index >30 kg/m^2^. Chronic hypertension and other hypertensive disorders of pregnancy (HDP) were defined by the nuMoM2b study using American College of Obstetricians and Gynecologist guidelines.^13^ Depression was measured by the Edinburgh Postnatal Depression Scale,^20^ which was dichotomized into scores <10 and 10+, given this is the typical threshold for identifying those who need further assessment for depressive symptoms.^21^ Resilience was measured using the Conner-Davidson Resilience Scale (range from 0 to 100, with higher values indicating greater resilience),^22^ stress with the Cohen Perceived Stress Scale (range from 0 to 40, with high scores indicating higher stress),^23^ anxiety with the trait subscale of the Spielberger Anxiety Inventory (range from 20 to 80, with higher scores indicating higher anxiety),^24^ and social support with the Multidimensional Scale of Perceived Social Support (range 12–84, with higher scores indicating greater support);^25^ all scales were administered prospectively in the nuMoM2b study. Diet was assessed using the Alternate Healthy Eating Index, a scale developed in 2002 based upon dietary recommendations to reduce chronic disease.^26^ Exercise was defined as reporting physical activity within the past month at their first study visit.

### Statistical analysis

The primary analysis employed logistic regression to assess the relationship between self-reported experiences of REC discrimination and the composite end-point of chronic hypertension and obesity. These two end-points were selected *a priori,* as they are the most prevalent risk factors for preeclampsia, but were combined into a composite as sample size limited our ability to estimate multivariate models for each outcome individually.

**FIG. 2.**
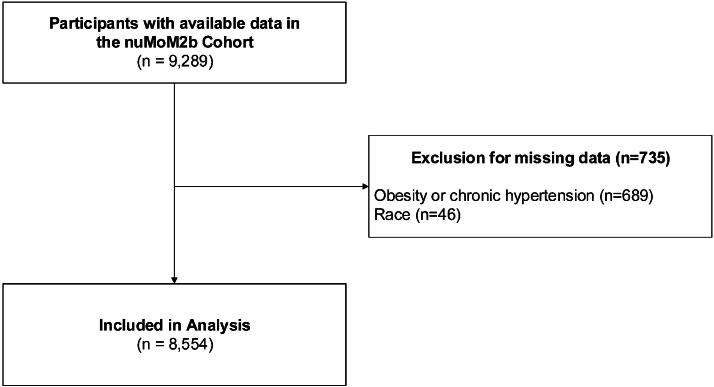
CONSORT diagram: Flowchart illustrating how we constructed our sample from the full dataset originally disseminated. Final analytic sample contains *n* = 8,554.

Variables with missing values were multiply imputed by chained equation using 30 imputations; imputation models were estimated using all variables considered for model inclusion prior to assessing for collinearity. Missing values for race (as a likely contributor to REC discrimination, our explanatory variable), chronic hypertension, and obesity (outcomes) were not imputed, and participants with missing values of these variables were excluded (Fig. 2). Collinearity was assessed based on a criterion of variance inflation factor >5, which no variables met, so all were included. For the explanatory variable, self-reported REC discrimination, high discrimination was defined as 3+ experiences of REC discrimination, low discrimination as 1–2 experiences, and no discrimination as 0 experiences based on scale use in prior research.^27^

Multivariable model variables were determined *a priori*, based on our theoretical model and community consultation. Those variables we thought might mediate the relationship between experiences of REC discrimination and obesity or chronic hypertension, such as stress and anxiety, were deliberately excluded from the multivariable model. Included variables by logic model category were age; psychosocial: resilience, social support; health behaviors: weekly drinks pre-pregnancy, Alternate Healthy Eating Index, smoking status pre-pregnancy, exercise within the prior month; and SDOH: percentage of poverty line, education level, birth outside of the United States, insurance status, employment status. Being foreign-born or previously living >1 year outside of the United States was assessed for interaction with REC discrimination.

Models were built by adding terms sequentially in groups based on the theoretical model (Fig. 1). Variable groups were added from most to least proximal in the theoretical model to chronic hypertension and obesity, yielding five models. These were adjusted as follows (categories defined above): (1) adjusted for age, (2) adjusted for age and psychosocial variables, (3) adjusted for age and health behaviors, (4) adjusted for age, psychosocial variables, and health behaviors, and (5) adjusted for age, psychosocial variables, health behaviors, and SDOH. Models were repeated with stratification by race and ethnicity; only the categories of non-Hispanic Black, Non-Hispanic White, and Hispanic had sufficient sample sizes, so those who identified otherwise were excluded for stratified analyses only.

An alpha level of 0.05 was selected *a priori* as statistically significant. All analyses were performed in R, version 4.2.1 (R Project for Statistical Computing) and Stata Statistical Software, version 17.0 (Statacorp, College Station, Texas). As the nuMoM2b dataset is a Limited Data Set, this study was determined to be exempt from review by the Duke University Health System Institutional Review Board. Patients participating in the nuMoM2b provided informed consent for their study data to be used for secondary analyses.

## Results

After excluding those with missing values for race and ethnicity, chronic hypertension, or obesity, our final analytic sample included 8,554 individuals. Among the cohort, 61.9% self-identified as non-Hispanic White, 16.5% as Hispanic, 13.2% as non-Hispanic Black, 4.1% as multiracial, 3.9% as Asian, 0.4% as Native Hawaiian, and 0.1% as American Indian. The average participant attended or completed college, was employed, commercially insured, and had an income greater than 200% of the poverty line (Table 1).

**Table 1.** Demographic and Clinical Characteristics Stratified by Self-Reported Race and Ethnicity

	Mean (standard deviation) or *N* (%)				*p*-Value
	Overall (*n* = 8,554)	Non-Hispanic White (*n* = 5,293)	Non-Hispanic Black (*n* = 1,127)	Hispanic (*n* = 1,412)	
Age (years)	27.0 (5.6)	28.2 (5.1)	23.4 (5.3)	24.8 (5.5)	<0.001
Education					<0.001
Less than HS grad	684 (8.0)	211 (4.0)	209 (18.5)	199 (14.1)	
HS grad or GED	996 (11.6)	391 (7.4)	286 (25.4)	256 (18.1)	
Some college	1,662 (19.4)	787 (14.9)	332 (29.5)	416 (29.5)	
Assoc/Tech degree	867 (10.1)	538 (10.2)	110 (9.8)	157 (11.1)	
Completed college	2,367 (27.7)	1,827 (34.5)	118 (10.5)	237 (16.8)	
Degree work beyond college	1,978 (23.1)	1,539 (29.1)	72 (6.4)	147 (10.4)	
Race and ethnicity					<0.001
Non-Hispanic White	5,293 (61.9)	5,293 (100.0)	0 (0.0)	0 (0.0)	
Non-Hispanic Black	1,127 (13.2)	0 (0.0)	1,127 (100.0)	0 (0.0)	
Hispanic	1,412 (16.5)	0 (0.0)	0 (0.0)	1,412 (100.0)	
American Indian	7 (0.1)	0 (0.0)	0 (0.0)	0 (0.0)	
Asian	335 (3.9)	0 (0.0)	0 (0.0)	0 (0.0)	
Native Hawaiian	31 (0.4)	0 (0.0)	0 (0.0)	0 (0.0)	
Multiracial	349 (4.1)	0 (0.0)	0 (0.0)	0 (0.0)	
Employed (missing = 1706)	5,383 (78.6)	3,844 (86.7)	436 (56.8)	687 (64.1)	<0.001
Insurance Status (missing = 56)					<0.001
Commercial	5,824 (68.5)	4,379 (83.1)	380 (34.5)	580 (41.3)	
Governmental	2,300 (27.1)	690 (13.1)	664 (60.2)	759 (54.0)	
Military	48 (0.6)	34 (0.6)	3 (0.3)	7 (0.5)	
Out of pocket	326 (3.8)	167 (3.2)	56 (5.1)	60 (4.3)	
Partnered (missing = 4)	8,072 (94.4)	5,153 (97.4)	935 (83.0)	1,298 (92.1)	<0.001
Born outside the U.S. (missing = 20)	1,088 (12.7)	229 (4.3)	58 (5.2)	542 (38.5)	<0.001
Household income as % of poverty line (missing = 1,557)					<0.001
>200%	4,892 (69.9)	3,799 (79.0)	211 (30.8)	453 (50.4)	
100–200%	1,010 (14.4)	581 (12.1)	151 (22.0)	191 (21.2)	
<100%	1,095 (15.6)	426 (8.9)	324 (47.2)	255 (28.4)	
Self-reported experiences of REC discrimination (missing = 262)					<0.001
0 experiences	6,380 (76.9)	4,507 (86.8)	613 (57.5)	875 (65.8)	
1–2 experiences	1,376 (16.6)	576 (11.1)	267 (25.0)	313 (23.6)	
3+ experiences	536 (6.5)	107 (2.1)	186 (17.4)	141 (10.6)	
Perceived Stress Scale (missing = 42)	12.8 (6.6)	11.9 (6.3)	15.2 (7.3)	13.8 (6.8)	<0.001
Edinburgh Postnatal Depression Scale Score ≥10 (missing = 225)	1,461 (17.5)	760 (14.6)	274 (25.9)	308 (22.5)	<0.001
Conner-Davidson Resilience Scale (missing = 423)	79.3 (11.7)	79.7 (11.1)	79.4 (13.6)	77.9 (12.5)	<0.001
Multidimensional Scale of Perceived Social Support (missing = 897)	74.4 (14.2)	75.7 (13.5)	69.8 (16.4)	73.1 (14.1)	<0.001
Spielberger Anxiety Inventory, trait subscale (missing = 1037)	33.8 (8.7)	33.3 (8.5)	35.5 (9.4)	34.6 (9.0)	<0.001
Alternate Healthy Eating Index (missing = 1311)	55.0 (12.5)	57.2 (12.3)	46.1 (10.2)	51.9 (11.1)	<0.001
Number of weekly drinks prior to pregnancy (missing = 697)	3.1 (5.7)	3.3 (5.2)	2.3 (4.7)	2.9 (7.9)	<0.001
Smoked prior to pregnancy (missing = 3)	1,539 (18.0)	893 (16.9)	306 (27.2)	205 (14.5)	<0.001
Exercised in past 4 weeks (missing = 1)	6,030 (70.5)	4,074 (77.0)	659 (58.5)	786 (55.7)	<0.001
Chronic hypertension	219 (2.6)	114 (2.2)	64 (5.7)	27 (1.9)	<0.001
Obesity (BMI ≥ 30)	1,899 (22.2)	1,006 (19.0)	422 (37.4)	330 (23.4)	<0.001
Preeclampsia (missing = 2)	764 (8.9)	431 (8.1)	147 (13.0)	128 (9.1)	<0.001

*p*-Values by analysis of variance for continuous variables and χ^2^ test for binary/categorical variables.

BMI, body mass index; HS, high school; GED, general educational development; REC, race, ethnicity, and color; U.S., United States.

When stratified by race and ethnicity, there were statistically significant differences in all demographic and clinical measures reported (Table 1; see Supplementary Table S1 for data from all racial and ethnic groups). Disparities followed a pattern with non-Hispanic White participants having the highest prevalence of characteristics considered health-promoting including being the most formally educated, most likely to be employed, of the highest income, and most likely to be commercially insured. For most variables, non-Hispanic Black participants had the highest prevalence of disadvantageous risk factors, while the prevalence of Hispanic participants with disadvantageous risk factors usually fell between non-Hispanic Black and non-Hispanic White participants. There were exceptions: Hispanic participants had the lowest resilience scores, the least exercise in the past month, the lowest smoking rates pre-pregnancy, and the lowest prevalence of chronic hypertension.

**FIG. 3.**
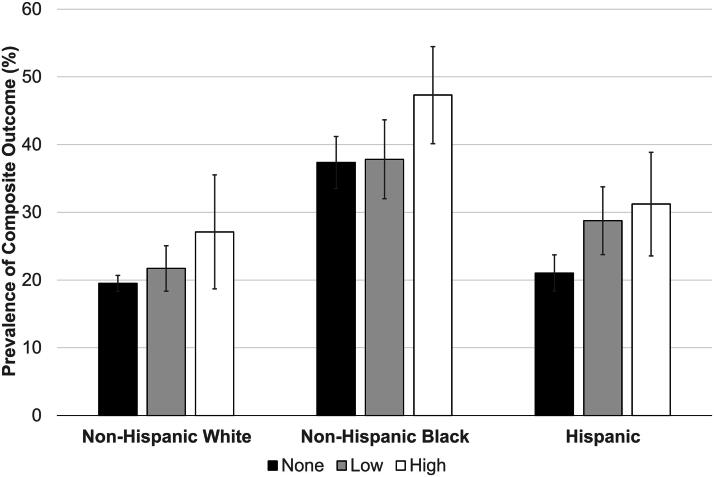
Bar graph depicting the prevalence of the composite of obesity and hypertension by experiences of racial, ethnic, and color-based (REC) discrimination, stratified by race and ethnicity group. Error bars represent 95% confidence intervals.

Over 75% of study participants reported no REC discrimination, and only 6.5% reported high REC discrimination. When stratified by race and ethnicity, 17.5% of non-Hispanic Black participants reported high REC discrimination, compared with 10.2% of Hispanic and 2.1% of non-Hispanic White participants. The overall prevalence of obesity and chronic hypertension was 22.2% and 2.6%, respectively. When combined into a composite outcome, prevalence was 23.1%. When stratified by REC discrimination, there was a positive relationship between REC discrimination and composite outcome prevalence. After further stratification by race and ethnicity, this positive relationship remained within subgroups; however, the composite outcome prevalence among non-Hispanic Black participants was higher than any other group for all levels of REC discrimination (Fig. 3).

The primary multivariable analysis involved sequential addition of potential confounding factors to a model of the composite outcome, as described in the methods (Table 2). We performed two joint tests for interaction between (1) being born outside of the United States or (2) having a history of living outside of the United States for more than a year and self-reported discrimination within the Hispanic subgroup, both of which were non-significant (*p*-values of 0.98 and 0.85, respectively). Therefore, these interaction terms were not included in our models.

**Table 2.** Odds Ratios and 95% Confidence Intervals of Composite (Obesity and/or Chronic Hypertension) among Those Reporting Low or High REC Discrimination Compared with Those Reporting no REC Discrimination

	Unadjusted	Model 1	Model 2	Model 3	Model 4	Model 5
Low discrimination	1.32 (1.15–1.51)	1.32 (1.15–1.51)	1.30 (1.14–1.49)	1.26 (1.10–1.44)	1.25 (1.09–1.43)	1.30 (1.13–1.50)
High discrimination	1.92 (1.59–2.32)	1.93 (1.60–2.33)	1.89 (1.56–2.28)	1.71 (1.41–2.08)	1.70 (1.40–2.06)	1.75 (1.43–2.14)
Model adjusts for:						
Age		✓	✓	✓	✓	✓
Psychosocial			✓		✓	✓
Health behavior				✓	✓	✓
SDOH						✓

Models performed with logistic regression.

High discrimination defined as those self-reporting 3 or more experiences of REC discrimination, low discrimination is defined as those self-reporting 1–2 experiences of REC discrimination.

Psychosocial variables: resilience, social support; health behavior variables: smoking prior to pregnancy, number of weekly drinks prior to pregnancy, diet (Alternate Healthy Eating Index), exercised in past 4 weeks; social determinants of health (SDOH) variables: born outside the United States, education, income, insurance type, poverty level.

In unadjusted analyses, high REC discrimination was associated with a higher risk of the composite outcome (odds ratio [OR] 1.92; 95% confidence interval [CI]: 1.59–2.32) as was low REC discrimination (OR 1.32; CI: 1.15–1.51) both when compared with those reporting no REC discrimination. The fully adjusted model (model 5) partially attenuated these estimates, estimating a 1.75 adjusted OR (aOR) for the composite outcome (95% CI 1.43–2.14) among those reporting high REC discrimination and a 1.30 aOR (95% CI 1.13–1.50) among those reporting low REC discrimination, after adjustment for psychosocial, health behavior, and SDOH variables. We then stratified these models by race and ethnicity (Table 3). The odds of the composite outcome were only statistically significant among the Hispanic subgroup, both among those reporting low (aOR 1.44, 95% CI 1.07–1.96) and high (aOR 1.54, 95% CI 1.03–2.32) REC discrimination when compared with those reporting no REC discrimination.

**Table 3.** Odds Ratios and 95% Confidence Intervals of Composite (Obesity and/or Chronic Hypertension) Among Those Reporting High and Low REC Discrimination Compared with Those Reporting No REC Discrimination, Stratified by Self-Reported Race and Ethnicity

	Unadjusted	Model 1	Model 2	Model 3	Model 4	Model 5
Non-Hispanic White						
Low discrimination	1.14 (0.92–1.41)	1.14 (0.92–1.41)	1.12 (0.91–1.39)	1.10 (0.88–1.36)	1.09 (0.88–1.36)	1.12 (0.90–1.39)
High discrimination	1.51 (0.97–2.33)	1.50 (0.97–2.32)	1.47 (0.95–2.28)	1.31 (0.84–2.05)	1.31 (0.84–2.05)	1.31 (0.83–2.09)
Non-Hispanic Black						
Low discrimination	1.03 (0.77–1.38)	0.91 (0.67–1.23)	0.91 (0.67–1.24)	0.91 (0.67–1.24)	0.92 (0.67–1.25)	0.90 (0.66–1.24)
High discrimination	**1.50 (1.08–2.09)**	1.07 (0.75–1.53)	1.06 (0.74–1.52)	1.04 (0.72–1.50)	1.03 (0.72–1.49)	1.05 (0.72–1.52)
Hispanic						
Low discrimination	**1.46 (1.09–1.95)**	**1.42 (1.06–1.90)**	**1.41 (1.05–1.89)**	**1.39 (1.03–1.86)**	**1.38 (1.03–1.86)**	**1.44 (1.07–1.96)**
High discrimination	**1.63 (1.11–2.41)**	**1.58 (1.07–2.33)**	**1.56 (1.05–2.31)**	**1.50 (1.01–2.23)**	**1.49 (1.00–2.21)**	**1.54 (1.03–2.32)**
Model adjusts for:						
Age		✓	✓	✓	✓	✓
Psychosocial			✓		✓	✓
Health behavior				✓	✓	✓
SDOH						✓

Bolded values indicate statistically significant results.

Models performed with logistic regression.

High discrimination defined as those self-reporting 3 or more experiences of REC discrimination, low discrimination is defined as those self-reporting 1–2 experiences of REC discrimination.

Check marks indicate which variable or variable groups were adjusted for in each model.

Psychosocial variables: resilience, social support; health behavior variables: smoking prior to pregnancy, number of weekly drinks prior to pregnancy, diet (Alternate Healthy Eating Index), exercised in past 4 weeks; social determinants of health (SDOH) variables: born outside the United States, education, income, insurance type, poverty level.

## Discussion

This secondary analysis of a diverse obstetric cohort of nulliparous patients demonstrates that obesity and chronic hypertension, commonly cited chronic conditions predisposing birthing people to preeclampsia, are associated with self-reported experiences of REC discrimination. Moreover, this relationship between REC discrimination and our composite outcome of obesity and chronic hypertension was attenuated when stratifying by race and ethnicity. This finding demonstrates that the increased odds of obesity and/or chronic hypertension are driven more strongly by differences in REC discrimination between racial and ethnic groups, rather than by variation in REC discrimination within racial and ethnic groups.

The persistent relationship within the Hispanic subgroup between REC discrimination and the composite outcome is notable, given the Hispanic subgroup is derived from an ethnic identity and includes individuals of all races. Given that the discrimination instrument asked specifically about racial, ethnic, and color-based discrimination, we might expect individuals of different races and colors within the Hispanic subgroup to have varying experiences of discrimination and biopsychosocial reactions to this discrimination. For this reason, the REC question may have been more effective at differentiating Hispanic individuals reporting extensive histories of racist experiences from those who have less extensive histories.

The link between racial and ethnic discrimination and adverse pregnancy outcomes is well-established; however, the majority of literature focuses on birth outcomes such as preterm labor and low birth weight, with scant research on maternal health outcomes.^28^ In a 2022 meta-analysis and systematic review, only one study was identified that included an assessment of the relationship between racial discrimination and HDP.^28^ Notably, this study also utilized nuMoM2b cohort data and identified an unadjusted 30% increased odds of HDP among non-Hispanic Black participants when compared with non-Hispanic White participants (95% CI 1.10–1.53).^29^ However, after adjusting for factors including both body mass index and medical co-morbidities (including chronic hypertension), the relationship was no longer significant. Similarly, another secondary nuMoM2b analysis also found a significant association between race and HDP, but concluded “modifiable risk-factors” such as obesity were more important than race, after adjusting for these variables in multivariable analysis.^30^ Given the current study’s findings suggesting a link between REC discrimination and obesity and chronic hypertension in this same cohort, as well as other studies demonstrating similar findings in non-obstetric cohorts,^9,10,31^ it is possible inclusion of these variables as confounders may underestimate the contributory role of racism in HDP.

Data suggests racism drives racial health disparities, acting multi-modally through structural, personally-mediated, and internalized racism to influence health outcomes.^32–34^ Although SDOH explain a portion of maternal health disparities, disparities persist even after accounting for these SDOH, and SDOH themselves are affected by racism.^16^ Despite these complex interactions,^12^ studies assessing disparities in maternal outcomes often adjust for conditions such as obesity and chronic hypertension, effectively treating these variables as confounding the effects of racism on health outcomes, rather than possible mediators in this pathway. By demonstrating the relationship between chronic health conditions and discrimination in an obstetric cohort, we aim to encourage obstetric researchers to recognize how chronic health conditions, such as obesity and chronic hypertension, may be on the causal pathway between racism and maternal health outcomes and therefore recognize the impact of the life course.

Although this study has important implications, its results need to be interpreted within the context of its limitations. Due to a limited number of outcome events, we analyzed a composite of hypertension and obesity, limiting our ability to parse the effect of discrimination on these two associated, but nonetheless distinct, conditions. The nuMom2b cohort is not nationally representative, and the cohort has a constellation of characteristics that make it lower-risk, on average, than the overall U.S. birthing population. Moreover, the Southeast, a region with some of the highest maternal morbidity and mortality rates in the United States,^35^ had no recruitment sites. The nuMom2b cohort is a cohort exclusively of nulliparous people, and the results may not generalize to parous people, whose outcomes may be influenced by events of prior pregnancies.

Measuring discrimination is limited by the ability of the instrument to accurately capture this experience.^36^ In this cohort, well over half of the non-Hispanic Black participants reported no experiences of discrimination. This seems incongruent with previous research documenting the experiences of Black people in the United States.^37^ This may be attributable to self-reporting and social desirability biases, as well as the sociopolitical context of the early 2010s, when participants were recruited. Additionally, self-reported experiences of REC discrimination only capture a portion of the impact of racism on health, not capturing the complete extent of structural, interpersonal, and internal racism. Similarly, any quantitative measure of discrimination is limited in capturing discrimination’s true impact. Moreover, assessing REC discrimination does not capture the impact of intersecting identities on health.^36^ How discrimination may alter health outcomes is complex, limiting our ability to assess this relationship through our measured psychosocial and socioeconomic variables. This complexity is evident in our purposeful exclusion of anxiety, depression, and stress from the adjusted models; while these variables may act as confounders of the relationship, they are also possible mediators between discrimination and health outcomes.^36^

Despite limitations, this analysis has numerous strengths. The nuMoM2b dataset has rich psychosocial data, including SDOH and psychosocial measures, which are rarely comprehensively collected. Although it is not a nationally representative cohort, the cohort has a lower-risk profile for chronic health conditions and preeclampsia, likely biasing toward the null. Therefore, these results are likely to underestimate the impact of REC discrimination on outcomes nationwide. While the instrument used to capture REC discrimination has limitations, it is adapted from the validated Experiences of Discrimination scale,^19^ one of the more commonly used assessments of discrimination in biomedical research.

### Health equity implications

Considering the limitations and strengths, these results suggest important implications for research and clinical practice. Health disparities in chronic conditions that are overrepresented in non-Hispanic Black, Hispanic, and other populations affected by discrimination should be recognized as related to the wide-reaching effects of racism. Factors like these chronic conditions, therefore, may need to be treated differently in obstetric research, recognizing them as possible mediators between racism and maternal health outcomes, and that their importance as mediators may differ in direction or magnitude among people of different racial or ethnic identities (e.g., in our analyses most pronounced in non-Hispanic Black individuals). Analyses evaluating the effects of racism on outcomes that perform multivariate adjustment need to be performed with clear consideration of whether the included variables are confounding factors between racism and outcome or are instead mediating factors. Our results also suggest that racial disparities in maternal health outcomes cannot be eliminated through interventions during the pregnancy period alone. Racism impacts people from birth,^34^ contributing to chronic conditions that put people at higher risk of poor maternal outcomes, such as preeclampsia, during subsequent pregnancies.^1,2^ For this reason, it is unlikely that focusing on pregnancy care alone will be sufficient to eliminate racial and ethnic disparities, although work on pregnancy care is also important. Erasing disparities in maternal morbidity and mortality will require sustained attention to the longitudinal impact of racism on the lives of birthing people, and on those pathways through which racism affects health over the life span.

## Abbreviations Used

aOR: Adjusted odds ratio
BIDHFL: Born in Durham, Healthy for Life
BMI: Body mass index
HDP: Hypertensive disorders of pregnancy
LATIN-19: Latinx Advocacy Team & Interdisciplinary Network for COVID-19
REC: Racial, ethnic, color-based
SDOH: Social determinants of health
