# Supplementary material for: Racial, Ethnic, and Color-Based Discrimination and Pre-Pregnancy Risk Factors for Preeclampsia Among Nulliparous Patients
Source: Health Equity. 2025 May 23;9(1):270–80. doi: 10.1089/heq.2024.0173 (PMC12171705; doi:10.1089/heq.2024.0173)
Supplement: Supplementary Table S1 [file heq.2024.0173_supplementary_table_s1.docx]

Supplemental Table 1: Extension of Demographic and Clinical Characteristics stratified by race and ethnicity, for race and ethnicity groups not included in manuscript body

|  | Overall  (N=8,554) | American Indian  (N=7) | Asian  (N=335) | Native Hawaiian  (N=31) | Multiracial  (N=349) |
| --- | --- | --- | --- | --- | --- |
|  | Mean (Standard Deviation) or N (%) | | | | |
| Age | 27.0 (5.6) | 25.0 (3.9) | 30.9 (4.7) | 27.2 (5.1) | 25.1 (6.2) |
| Education |  |  |  |  |  |
| Less than HS grad | 684 (8.0) | 1 (12.5) | 4 (1.1) | 1 (3.1) | 65 (17.2) |
| HS grad or GED | 996 (11.6) | 1 (12.5) | 10 (2.7) | 5 (15.6) | 53 (14.1) |
| Some college | 1,662 (19.4) | 4 (50.0) | 36 (9.9) | 6 (18.8) | 91 (24.1) |
| Assoc/Tech degree | 867 (10.1) | 1 (12.5) | 31 (8.5) | 4 (12.5) | 33 (8.8) |
| Completed college | 2,367 (27.7) | 0 (0.0) | 113 (31.0) | 9 (28.1) | 75 (19.9) |
| Degree work beyond college | 1,978 (23.1) | 1 (12.5) | 170 (46.7) | 7 (21.9) | 60 (15.9) |
| Employed (missing = 1706) | 5,383 (78.6) | 4 (80.0) | 223 (76.6) | 21 (84.0) | 193 (67.0) |
| Insurance Status (missing = 56) |  |  |  |  |  |
| Commercial | 5,824 (68.5) | 4 (50.0) | 308 (84.4) | 20 (62.5) | 194 (51.9) |
| Governmental | 2,300 (27.1) | 3 (37.5) | 38 (10.4) | 10 (31.2) | 154 (41.2) |
| Military | 48 (0.6) | 0 (0.0) | 1 (0.3) | 0 (0.0) | 3 (0.8) |
| Out of Pocket | 326 (3.8) | 1 (12.5) | 18 (4.9) | 2 (6.2) | 23 (6.1) |
| Partnered (missing = 4) | 8,072 (94.4) | 8 (100.0) | 360 (98.9) | 31 (96.9) | 344 (91.2) |
| Born Outside the U.S. (missing = 20) | 1,088 (12.7) | 0 (0.0) | 240 (66.3) | 12 (37.5) | 29 (7.7) |
| Household Income as % of Poverty Line (missing = 1557) |  |  |  |  |  |
| >200% | 4,892 (69.9) | 2 (33.3) | 284 (86.1) | 18 (64.3) | 158 (54.3) |
| 100%-200% | 1,010 (14.4) | 1 (16.7) | 29 (8.8) | 2 (7.1) | 61 (21.0) |
| <100% | 1,095 (15.6) | 3 (50.0) | 17 (5.2) | 8 (28.6) | 72 (24.7) |
| Self-reported Experiences of REC Discrimination (missing = 262) |  |  |  |  |  |
| 0 experiences | 6,380 (76.9) | 3 (42.9) | 178 (51.4) | 15 (46.9) | 202 (56.0) |
| 1-2 experiences | 1,376 (16.6) | 2 (28.6) | 115 (33.2) | 12 (37.5) | 108 (29.9) |
| 3+ experiences | 536 (6.5) | 2 (28.6) | 53 (15.3) | 5 (15.6) | 51 (14.1) |
| Perceived Stress Scale (missing = 42) | 12.8 (6.6) | 18.2 (5.9) | 12.6 (5.6) | 14.8 (6.9) | 14.1 (7.0) |
| Edinburgh Postnatal Depression Scale Score ≥10 (missing = 225) | 1,461 (17.5) | 4 (50.0) | 54 (15.3) | 8 (25.8) | 65 (17.8) |
| Conner-Davidson Resilience Scale (missing = 423) | 79.3 (11.7) | 74.9 (18.6) | 76.9 (11.1) | 79.8 (9.9) | 79.6 (11.1) |
| Multidimensional Scale of Perceived Social Support (missing = 897) | 74.4 (14.2) | 64.1 (25.4) | 75.1 (12.5) | 72.8 (17.3) | 72.4 (14.5) |
| Spielberger Anxiety Inventory, trait subscale (missing = 1037) | 33.8 (8.7) | 37.7 (5.6) | 33.7 (7.8) | 35.4 (8.6) | 34.5 (8.4) |
| Alternate Healthy Eating Index (missing = 1311) | 55.0 (12.5) | 52.5 (17.5) | 61.2 (9.5) | 52.5 (12.0) | 51.7 (12.4) |
| Number of weekly drinks prior to pregnancy (missing = 697) | 3.1 (5.7) | 2.1 (3.3) | 2.2 (4.5) | 1.9 (2.4) | 3.4 (5.7) |
| Smoked prior to pregnancy (missing = 3) | 1,539 (18.0) | 3 (37.5) | 24 (6.6) | 11 (34.4) | 105 (27.9) |
| Exercised in past four weeks (missing = 1) | 6,030 (70.5) | 5 (62.5) | 256 (70.1) | 25 (78.1) | 261 (69.2) |
| Chronic hypertension | 219 (2.6) | 0 (0.0) | 3 (0.9) | 1 (3.2) | 10 (2.8) |
| Obesity (BMI ≥ 30) | 1,899 (22.2) | 4 (50.0) | 28 (7.8) | 11 (34.4) | 106 (28.6) |
| Pre-eclampsia (missing = 2) | 764 (8.9) | 1 (14.3) | 16 (4.7) | 3 (9.7) | 39 (11.0) |
| HS = High School, GED = General Educational Development, REC = Race, Ethnicity, and Color, BMI = Body mass index, U.S. = United States | | | | | |
