# Supplementary material for: Racial, Ethnic, and Color-Based Discrimination and Pre-Pregnancy Risk Factors for Preeclampsia Among Nulliparous Patients
Source: Health Equity. 2025 May 23;9(1):270–80. doi: 10.1089/heq.2024.0173 (PMC12171705; doi:10.1089/heq.2024.0173)
Supplement: Supplementary Table S2 [file heq.2024.0173_supplementary_table_s2.docx]

Supplemental Table 2: Local Community Organizations Represented During Meetings with Born in Durham, Healthy for Life (BIDHFL) and LATIN-19

| **BIDHFL Meeting** | **LATIN-19 Meeting** |
| --- | --- |
| Born in Durham, Healthy for Life | Duke Health |
| Breastfeed Durham | UNC Medical Center |
| Center for Child & Family Health | North Carolina Central University |
| Duke Family Medicine Center | El Futuro |
| Duke Division of Maternal Fetal Medicine | Immersion for Spanish Language Acquisition (ISLA) |
| Duke Department of Obstetrics and Gynecology | El Centro Hispano |
| Durham County Department of Public Health | La Semilla |
| MAAME Inc. | NC Counts Coalition |
| The Precious Cargo Foundation | Durham Public Schools |
|  | Samaritan Health Center |
|  | Durham County Government and Health Department |
|  | Wake County Government and Health Department |
|  | Orange County Health Department |
|  | Carolina Complete Health |
|  | WakeMed |
|  | American Heart Association, NC chapter |
|  | North Carolina Department of Health and Human Services |
|  | Duke School of Medicine |
|  | Duke School of Nursing |
|  | Duke School of Engineering |
|  | Unaffiliated community members |
